# Bridging the Gap: The Need to Implement Dietary Guidance to Address Cardiovascular Health

**DOI:** 10.3390/nu16132125

**Published:** 2024-07-03

**Authors:** Alison G. M. Brown, Samantha Adas, Janet de Jesus, Nicole Farmer, Rachel Fisher, Charlotte A. Pratt

**Affiliations:** 1 National Heart Lung and Blood Institute, Division of Cardiovascular Sciences, U.S. Department of Health and Human Services, Bethesda, MD 20892, USA; prattc@nhlbi.nih.gov; 2 Office of Nutrition Research, National Institutes of Health, U.S. Department of Health and Human Services, Bethesda, MD 20892, USA; samantha.adas@nih.gov; 3 Office of Disease Prevention and Health Promotion, U.S. Department of Health and Human Services, Rockville, MD 20852, USA; janet.dejesus@hhs.gov (J.d.J.); rachel.fisher@hhs.gov (R.F.); 4 Translational Biobehavioral and Health Disparities Branch, National Institutes of Health Clinical Center, U.S. Department of Health and Human Services, Bethesda, MD 20892, USA; nicole.farmer@nih.gov

**Keywords:** cardiovascular diseases, Mediterranean diet, DASH diet, dietary guidelines, nutrition policy, implementation science, Food is Medicine

## Abstract

Cardiovascular disease (CVD) is the leading cause of death in the U.S. and globally. Research demonstrates that diet is a leading contributor to the development of CVD, its prevention and management, and the overall promotion of cardiovascular health. This article describes the current state of the evidence, including research on the DASH and Mediterranean diets to promote cardiovascular health and prevent CVD. The article suggests approaches to implement evidence-based diets and federal dietary guidance to promote the adoption and integration of these interventions in both community and clinical settings. It highlights the current U.S. federal interest in “Food is Medicine” and its importance in addressing diet-related chronic diseases and promoting cardiovascular health.

## 1. Introduction

Cardiovascular disease (CVD) remains a leading cause of mortality in the United States (U.S.), imposing a substantial economic burden on the nation. The average annual direct and indirect cost of CVD in the United States was an estimated USD 422.3 billion in the period from 2019 to 2020 [1,2]. By 2035, medical spending on CVD is projected to be USD 749 billion according to the American Heart Association (AHA) [3]. Research has found that optimizing health behaviors and factors, including diet and nutrition, can improve cardiovascular health (CVH) and reduce the risk for CVD. Despite this, adherence to the *Dietary Guidelines for Americans* (*Dietary Guidelines*) and the quality of dietary intake remain low across the United States [4]. Health disparities rooted in race, ethnicity, socioeconomic status, and geographic location significantly impact diet quality and health outcomes, increasing risk factors associated with CVD [5].

In 2022, the AHA released Life’s Essential 8, which underscores eight health factors and behaviors that are paramount to maintaining and improving CVH [6,7]. These include Eat Better, Be More Active, Quit Tobacco, Get Healthy Sleep, Manage Weight, Control Cholesterol, Manage Blood Sugar, and Manage Blood Pressure. The first of Life’s Essential 8, Eat Better, highlights nutrition as a key factor in maintaining CVH and preventing CVD. The AHA recommends creating a healthy eating pattern by incorporating fruits, vegetables, whole grains, beans, legumes, nuts, lean protein-rich foods including plant-based proteins in one’s diet, which is reflected in the Dietary Approaches to Stop Hypertension (DASH) dietary plan and Mediterranean diet. In addition, the AHA encourages cooking at home and learning to read nutrition labels, particularly to be mindful of salt in foods and to empower individuals to make healthy choices.

Current research support healthy dietary patterns described in the *Dietary Guidelines* along with the DASH and Mediterranean dietary patterns, which are shown to improve CVH [4]. However, Life’s Essential 8 scores among U.S. adults show that diet is one of the lowest score components, with significant gender and ethnicity disparities present [8,9,10]. Understanding how to best implement these federally supported dietary recommendations across the U.S., while addressing barriers that disproportionately affect diverse groups, is needed to equitably improve CVH in the U.S. population and reduce the economic burden [11].

The U.S. government continues to include nutrition as a vital factor in achieving optimal health and disease prevention, as depicted in the National Strategy on Hunger, Nutrition, and Health [12]. The strategy also highlights the importance of diversity in nutrition research, which is vital to improving representation from groups typically underrepresented in biomedical research. Growing efforts in Food is Medicine also present opportunities to address nutrition’s role in CVH among other diet-related chronic diseases across multiple sectors, including the community and medical settings, along with policy implementation [13,14].

This review highlights two featured dietary patterns in the *Dietary Guidelines*, the DASH and the Mediterranean diet, and provides suggestions for implementing dietary guidance to address CVH. Additionally, the review delves into new federal efforts and opportunities that are underway to improve nutrition for all Americans.

## 2. Current Evidence: Dietary Approaches to Stop Hypertension (DASH) and Mediterranean Dietary Patterns

### Subsection

The DASH eating plan and the Mediterranean diet are two widely studied evidence-based dietary patterns to address CVD included in the Dietary Guidelines. DASH has been extensively studied in different populations with results demonstrating effectiveness in hypertension control. Nearly half of U.S. adults and an estimated 1.3 billion adults globally have hypertension. Thus, DASH is a key target for interventions related to hypertension and CVD risk prevention and management [15,16]. It encourages the consumption of fruits, vegetables, whole grains, low-fat dairy and of reduced amounts of sodium, saturated fat, and cholesterol. Its effects in controlling hypertension have been observed in African Americans and other populations, and it has the potential to reduce CVD risk and address CVD disparities [17]. For example, in the trial, participants on the DASH diet showed significantly lower systolic (SBP) and diastolic blood pressure (DBP) (by 5.5 and 3.0 mmHg, respectively) than those on the control diet (*p* < 0.001 for each), even without reduced sodium intake and weight loss [11]. Those with hypertension lowered SBP and DBP by 11.4 mmHg and 5.5 mmHg, respectively. Comparable findings have been demonstrated, with additional improvements in blood pressure with reductions in dietary sodium [18]. For example, Black participants lowered their systolic blood pressure by 12.6 mmHg compared to 9.5 mmHg for others when on the DASH diet with a low sodium level (1500 mg/day) [12]. In addition to the effect on blood pressure, adherence to the DASH dietary pattern is associated with reductions in total blood cholesterol, blood glucose, and BMI [19,20,21].

Evidence for the benefits of the Mediterranean diet originated from an ecological evaluation of multi-country dietary patterns among cohorts of men, with the dietary pattern from Greece serving as the representative Mediterranean pattern [22]. Since this initial study, benefits from the Mediterranean diet have been reported across many countries [23,24,25]. Although the diet may vary across countries, fundamentally the dietary pattern includes an emphasis on the consumption of unsaturated fat (principally from olive oil), vegetables, legumes, nuts, fruits, fish, and whole grains, while limiting saturated fat (with red meat as a main source), refined starch, and sugar-sweetened beverages or foods [26,27,28]. As a result of regional and country food cultures, variations in the recommendations of the Mediterranean diet occur within the dairy food group, with some Mediterranean diets limiting dairy, while others encouraging yogurt and cheese. Although the Mediterranean diet does not provide specific recommendations on sodium intake, the emphasis on the consumption of unprocessed foods may inherently reduce the sodium intake [29]. Moreover, DASH–Mediterranean dietary pattern interventions or interventions comparing the DASH and the Mediterranean patterns have shown a beneficial impact on the prevention of cognitive decline, and the reduction of sodium intake with both patterns have contributed to reductions in hypertension, and cardiovascular events [30,31,32]. Table 1 provides a comparison of the Mediterranean and DASH patterns based on featured components of the dietary patterns for CVH, CVH factors from the Life’s Essential 8 score, and specific emphasized nutrients within each pattern related to CVD. The patterns featured in Table 1 contribute to the strong conclusion by the 2020 Dietary Guidelines Advisory Committee, that stated, “ Strong and consistent evidence demonstrates that dietary patterns associated with decreased risk of cardiovascular disease are characterized by higher consumption of vegetables, fruits, whole grains, low-fat dairy, and seafood, and lower consumption of red and processed meat, and lower intakes of refined grains, and sugar-sweetened foods and beverages relative to less healthy patterns. Additionally, research that includes specific nutrients in their description of dietary patterns indicate that patterns that are lower in saturated fat, cholesterol, and sodium and richer in fiber, potassium, and unsaturated fats are beneficial for reducing cardiovascular disease risk [33].

## 3. Need for Implementation of Evidence-Based Diets

Despite the preponderance of scientific evidence on the importance of diet for the prevention, management, and treatment of CVD, there is a gap in implementing the science into clinical practice and community settings. Gaps in the adoption of these evidence-based interventions signal the need to advance implementation science in nutrition research to better understand the barriers and facilitators to adopting and maintaining healthy dietary behaviors [61]. Implementation science is defined as “the scientific study of methods to promote the systematic uptake of research findings and other evidence-based practices into routine practice, to improve the quality and effectiveness of health services” [62]. Implementation science also provides the opportunity to engage the clinical and community workforce to enhance the uptake and communication of evidence-based diets to the patients and communities that need it the most [63,64]. Enhancing implementation science in nutrition would provide the opportunity to understand how best to promote the reach, adoption, adaptation, and sustainability of evidence-based diets, ultimately contributing to improved CVH across a variety of diverse populations, environments, and settings—ranging from health care to public health and community-based organizations. Specifically, hybrid effectiveness–implementation science research is needed to promote adherence to diets such as the DASH and Mediterranean dietary patterns to control hypertension and other CVD risk factors [63] in multiple settings, geographic regions, and populations.

The strategy of addressing CVD through diet and nutrition involves multi-level interventions for its dissemination and implementation. This, in part, is because how people access, select, and consume foods is often influenced by multiple levels of the socio-ecological framework (e.g., individual, interpersonal, organizational, community, societal) [65]. Dissemination and implementation rests on the idea that study findings and interventions are understood across multiple socio-ecological levels. Conceptual or theoretical frameworks provide systematic and pragmatic guidance for planning, managing, and evaluating research and have the potential to aid in understanding how study findings or interventions can be disseminated and implemented at multiple socio-ecological levels.

Several frameworks and models have been proposed or used to guide dissemination and implementation research [66]. Within dietary or diet-related interventions, implementation frameworks have served as guidelines for planning food-related policies, obesity prevention programs, and food security interventions [67,68,69]. Among these frameworks, RE-AIM (Reach, Effectiveness, Adoption, Implementation, and Maintenance) has been cited extensively and consistently within the scientific literature during the past 20 years in over 2800 scientific publications [70]. RE-AIM is a cross-cultural, cross-topic framework that focuses on the design, dissemination, and implementation process [70]. Developed to address the issue of how to translate scientific knowledge into practice within community and clinical settings, RE-AIM has served as a model for the design and implementation of effective interventions to impact public health and address health equity [70,71].

Table 2 illustrates examples of how the RE-AIM framework can be adapted to implement dietary guidance to improve CVH. Specifically, dietary interventions could be targeted at populations with poor CVH (Reach), including low socioeconomic status populations, rural populations, and racial and ethnic minorities. Documentation of intervention outcomes (Effectiveness) should accompany dietary interventions including data on social determinants of health. Adoption of dietary guidance could consider contextual factors, including settings and cultural differences associated with food choices. The assessment of dose and fidelity of the intervention and participant skills and ability to implement knowledge gained from the intervention (Implementation), as well as strategies to sustain and adhere to dietary guidance interventions are needed (Maintenance).

The Biden–Harris Administration’s National Strategy on Hunger, Nutrition, and Health highlights the importance of implementing research findings to reduce the burden of chronic disease in the U.S. and outlines actions that the federal government will take to reach the goal of ending hunger and reducing diet-related diseases by 2030 [12]. Pillar 3 of the strategy recommends empowering all consumers to make and have access to healthy choices. From the foundation of nutrition research and science-based nutrition guidance to all federal nutrition efforts that promote health and chronic disease prevention, nutrition education and communication are essential to support consumers in making healthy choices by providing actionable guidance and tools. There is opportunity for multiple agencies to collaboratively strengthen the focus on public education to implement healthier food and beverage choices as part of a broader effort to reduce the risk for chronic diseases, including CVD. The *Dietary Guidelines* is the mainstay for a national nutrition campaign to boost awareness of healthy eating recommendations and support and empower all Americans in making healthy food choices, including those to prevent CVD. Communities may be faced with conflicting nutrition information and counterproductive food industry marketing strategies that target children, teens, and communities of color with proposals of unhealthy foods, contributing to CVD risk [72]. In addition, there is widespread misinformation about the scientific basis, transparency, and relevance of the *Dietary Guidelines* for the U.S. population that threatens its credibility within the scientific community and the lay public [73].

Mass media and public education campaigns can be highly effective at increasing awareness and achieving individual- and population-level behavior changes [74]. Because diet-related behavioral change is multifactorial and complex, efforts to change dietary behaviors should employ multidisciplinary, multi-dimensional approaches to effectively reach the public and improve nutrition. A national campaign, as outlined by the Biden–Harris Administration, could reach a wide range of people across the U.S. with consistent messages while providing culturally tailored nutrition information. Such a unified campaign would potentially more consistently convey nutrition advice to promote CVH and prevent chronic disease, combat misinformation and messages promoting unhealthy foods, and meet the needs of diverse communities, particularly those most at risk for CVD and other chronic diet-related conditions.

## 4. Food Is Medicine as an Opportunity to Address Cardiovascular Disease

Recognizing that more efforts are needed to address CVD risk and other diet-related chronic diseases, there is growing interest from both the public and the private sector in Food is Medicine interventions. Better integration of nutrition and health, including through Food is Medicine approaches, is another key pillar of the Biden–Harris Administration’s National Strategy on Hunger, Nutrition, and Health [12].

Food is Medicine strategies have the potential to reduce barriers to healthy eating and improve the implementation of healthy food patterns, including the DASH and Mediterranean diets, identified in the *Dietary Guidelines*, that can reduce the CVD risk [4]. HHS describes Food is Medicine as a broad range of approaches that promote optimal health and healing and reduce disease burden by providing nutritious food—in conjunction with human services, education, and policy change—through collaboration at the nexus of healthcare and community. These approaches are increasingly present across many communities and systems and include a spectrum of food-based interventions such as medically tailored meals or groceries, produce prescriptions, and nutrition incentives that can be integrated into healthcare for patients with specific health conditions or unmet social needs [75,76]. These food-based interventions that target those with CVD or at risk for CVD provide an opportunity to implement evidenced-based diets on a broader scale for greater public health impact.

Unmet health-related social needs such as housing, food, and transportation are significant contributors to poor health and have an adverse impact on CVD [77]. In a USDA study, the prevalence of CVD was six times higher in households with very low food security compared to food-secure households [78]. Research suggests that programs that address people’s social needs can promote direct reductions in healthcare costs. Recently, an evaluation of the North Carolina Department of Health and Human Services’ Healthy Opportunities Pilot study demonstrated that its program to address unmet social needs, including Food is Medicine interventions, resulted in a USD 85 reduction in medical costs per beneficiary per month [79].

In 2023, HHS developed a Food is Medicine initiative in response to a congressional directive to unify and advance collective action [80]. This directive called for the Secretary of HHS, in consultation with other federal agencies, to develop and implement a federal strategy to reduce nutrition-related chronic diseases and food insecurity to improve health and racial equity in the U.S. This includes diet-related research and programmatic efforts that will increase access to Food is Medicine programs and benefits. As part of this initiative, HHS is working collaboratively with federal partners and external organizations and communities to develop resources that can be used to advance Food is Medicine approaches across the U.S.

Overall, novel implementation research on dietary guidance is needed to address CVH and promote health equity. In Table 3 below, we provide selected examples of future research and strategies needed to address CVD.

## 5. Conclusions

The significant burden of CVD in the United States warrants a collaborative and concerted effort to address this national concern. As a key factor in shaping CVH, the implementation of evidenced-based diets—such as the DASH and Mediterranean dietary patterns—and of recommendations in the *Dietary Guidelines* is needed to address the diet-related chronic diseases that plague our nation, including CVD. The growing interest in Food is Medicine efforts provides promise for innovative and collective national action, involving multiple governmental agencies and a variety of other sectors. Future research is needed to evaluate the impact of Food is Medicine approaches to address CVD, but also studies using conceptual frameworks in the implementation of evidence-based dietary guidance, behavioral economic strategies to make the healthy choice the easy choice, and other efforts that address nutrition insecurity. Overall, expansive and transformative change is needed to address diet-related diseases that gravely impact the U.S. population, including the application of evidence-based diets through Food is Medicine strategies and implementation science to advance CVH and achieve a broader public health impact.

## Figures and Tables

**Table 1:** Cardiovascular health benefits of the Mediterranean and DASH dietary patterns.

Dietary Pattern	Featured Components of the Dietary Pattern	CVH Factors Commonly Impacted Using Non-Dietary American Heart Association Life’s Essential 8 Score Components [34]	Dietary Specific Nutrients Related to CVD
Mediterranean Diet	Emphasized: Vegetables; Fruits; Whole grains; Legumes; Nuts and seeds; Poultry; Fish and seafood (fatty); Extra-virgin olive oil as source of monounsaturated fat (MUFA). Limited: Dairy; Meat; Sugar-sweetened beverages; Commercial bakery goods, sweets, and pastries.	Blood lipids [35,36,37,38] Blood pressure [35,36,37,38] Weight loss [35,36,37,38] Blood glucose [39,40,41] Physical activity [42,43,44] Tobacco use [45,46] Sleep [47,48,49]	Increase of: Fiber; Polyphenols; Polyunsaturated Fats; Monounsaturated fats. Reduction of: Saturated fat.
Dietary Approaches to Stop Hypertension (DASH)	Emphasized: Vegetables; Fruits; Whole grains; Legumes; Nuts and seeds; Low-fat dairy. Limited: Saturated Fat; Sodium **; Fatty meats; Refined grains; Added sugars; Alcohol (≤2 drinks per day).	Blood lipids [50] Blood pressure [17,18,50,51] Weight [18,51] Blood sugar [52,53,54,55] Physical activity [56] Tobacco use [57,58] Sleep [55,59,60]	Increase of: Fiber; Polyphenols; Polyunsaturated Fats; Potassium. Reduction of: Sodium; Saturated fat.

** sodium levels within DASH Trial were set at 3000 mg per day, sodium levels within DASH–Sodium Trial compared 3000 mg, 2400 mg, and 1500 mg per day, with lower-dose effect found on blood pressure outcome.

**Table 2:** Adaptation of the RE-AIM framework to implement dietary guidance with the goal of improving cardiovascular health.

RE-AIM	
Reach	Develop strategies to target and work with populations with poor CVH. Focus interventions on addressing food insecurity, enhancing access to affordable and healthy foods, creating awareness of the *Dietary Guidelines*, and promoting health equity and CVH.
Effectiveness	Assess the impact of a dietary guidance intervention on outcomes (e.g., Life’s Essential 8 outcomes). Include outcomes such as social needs or risks, quality of life, and economics. Report on the heterogeneity of effects, including race and ethnicity, sex and gender differences. Include positive and null outcomes.
Adoption	Understand the contextual factors related to dietary guidance adoption, including the influence of settings (e.g., home, work, school, neighborhood), cultures, and social determinants of health factors. Develop procedures, tools, and methods to enhance adoption of dietary guidance. Assess barriers to adoption of dietary guidance and develop solutions to encourage adoption.
Implementation	Promote strategies to deliver dietary guidance interventions. For example, adaptation of the DASH or Mediterranean dietary patterns for different populations and cultures and label reading. Assess fidelity of intervention delivery, intervention dose, duration, and frequency, client satisfaction, implementation, cost, and impact on social determinants of health factors.
Maintenance	Promote strategies to sustain and adhere to dietary guidance interventions. Assess the extent to which individual and communities change behaviors and sustain those behaviors over time (e.g., 6 months or more post intervention). Assess institutionalization of program policies.

**Table 3:** Opportunities for future research and strategies to implement evidence-based dietary guidance.

Topic Areas	Selected Examples of Implementation Research and Strategy Needs
Implementation of *Dietary Guidelines* and Evidence-Based Dietary Recommendations	Research that uses conceptual frameworks such as the RE-AIM to address the implementation of the U.S. (and other) *Dietary Guidelines* to improve CVH with the goal of addressing CVH and health disparities. Assess the implications of social determinants of health, cultural food choices, access to and availability of healthy food choices for dietary guidance and dietary interventions to promote CVH. Research that examines the impact of cultural adaptations of dietary patterns (e.g., DASH and Mediterranean) to enhance the effectiveness of evidence-based diets to address CVD disparities.
Food and Nutrition Insecurity	Research that explores the impact of aligning foods provided in our emergency food system (e.g., food banks) with evidence-based dietary guidance (e.g., DASH, Mediterranean diet) in order to promote CVH among those experiencing food and nutrition insecurity.
Behavioral Economics in Food Retail Settings	Research to explore how various behavioral economic strategies (e.g., product price, placement, and promotion) impact consumer behavior in food retail environments, including online grocery shopping. Enhanced strategies to make the “healthy choice the easy choice” for all consumers in a variety of food retail environments.
Food is Medicine	Research that would use “Food is Medicine” approaches to address CVH outcomes and exploring which interventions, at what dose, and for what population are most effective at addressing CVD outcomes, including populations with health disparities such as low-income, racial, and ethnic minority populations and rural communities. Identification and adoption of consistent priority metrics that could be incorporated into research design and practice, making it easier to compare studies. Research on the cost-effectiveness of interventions as well as data on the wide spillover effects of Food is Medicine interventions (e.g., household and community resilience, improved mental health and economic stability).
